# ADAR1 Editing and its Role in Cancer

**DOI:** 10.3390/genes10010012

**Published:** 2018-12-25

**Authors:** Li-Di Xu, Marie Öhman

**Affiliations:** Department of Molecular Biosciences, The Wenner-Gren Institute, Stockholm University, Svante Arrhenius väg 20C, 106 91 Stockholm, Sweden; lidi.xu@su.se

**Keywords:** ADAR1, adenosine deamination, RNA editing, cancer, innate immunity

## Abstract

It is well established that somatic mutations and escape of immune disruption are two essential factors in cancer initiation and progression. With an increasing number of second-generation sequencing data, transcriptomic modifications, so called RNA mutations, are emerging as significant forces that drive the transition from normal cell to malignant tumor, as well as providing tumor diversity to escape an immune attack. Editing of adenosine to inosine (A-to-I) in double-stranded RNA, catalyzed by adenosine deaminases acting on RNA (ADARs), is one dynamic modification that in a combinatorial manner can give rise to a very diverse transcriptome. Since the cell interprets inosine as guanosine (G), A-to-I editing can result in non-synonymous codon changes in transcripts as well as yield alternative splicing, but also affect targeting and disrupt maturation of microRNAs. ADAR-mediated RNA editing is essential for survival in mammals, however, its dysregulation causes aberrant editing of its targets that may lead to cancer. ADAR1 is commonly overexpressed, for instance in breast, lung, liver and esophageal cancer as well as in chronic myelogenous leukemia, where it promotes cancer progression. It is well known that ADAR1 regulates type I interferon (IFN) and its induced gene signature, which are known to operate as a significant barrier to tumor formation and progression. Adding to the complexity, ADAR1 expression is also regulated by IFN. In this review, we discussed the regulatory mechanisms of ADAR1 during tumorigenesis through aberrant editing of specific substrates. Additionally, we hypothesized that elevated ADAR1 levels play a role in suppressing an innate immunity response in cancer cells.

## 1. Introduction

Editing of adenosine-to-inosine (A-to-I) in double stranded RNA [1,2,3], a common post-transcriptional modification in mammals, is mediated through adenosine deaminases acting on RNA (ADARs). In the ADAR family, ADAR1 [4,5] and ADAR2 [6] are the two catalytically active enzymes that are expressed in many tissues. ADAR1 is more ubiquitously expressed while most specific substrates for ADAR2 are in the brain, even though recent work has found the highest expression of ADAR2 in the arteries [7]. ADAR3 is editing incompetent and mainly expressed in the brain, but has been shown to inhibit ADAR1 and ADAR2 editing [8,9].

Inosine is interpreted as guanosine during base-pairing. Therefore, editing can lead to codon changes with the consequence of altered protein function, alternative splicing or affect targeting and maturation of microRNAs, depending on where it happens. It has been shown in several studies that the transcriptomic as well as the proteomic diversity introduced by A-to-I editing is exploited by tumor cells to promote cancer progression [10,11,12]. Acute myeloid leukemia (AML) was the first tumor type where altered mRNA editing was shown to be connected to the disease [13]. This study showed elevated editing with the consequence of increased intron retention in the transcript coding for protein tyrosine phosphatase, non-receptor type 6 (PTPN6) in patients with AML. Another early study showed dysregulation of editing in ADAR substrates in malignant gliomas [14]. Here, a reduced ADAR2 activity decreased the editing ratio at the Q/R site in subunit 2 of the ionotropic AMPA glutamate receptor (*GRIA2*, also known as *GluA2* and *GluR-B*) transcript, which was shown to be associated with tumor progression [14]. Since then, aberrant A-to-I editing on specific transcripts and their association with cancer prognosis or cancer metastasis have been demonstrated in many different cancer types.

Although both ADAR1 and ADAR2 have been shown to play roles in tumorigenesis, more editing events regulated by ADAR1 have been associated with cancer development, primarily due to more abundant expression of ADAR1 [11] and its unique features. One feature is that *Adar1* localizes on chromosome 1 arm q, a region that is frequently amplified in cancer [15]. Another feature is that ADAR1 has two isoforms, the interferon (IFN) inducible full-length ADAR1^p150^ and a shorter and constitutively expressed ADAR1^p110^ [16,17,18]. Both isoforms shuttle between nucleus and cytoplasm [19]; however, ADAR1^p110^ is constitutively expressed and localizes mainly to the nucleus, consistent with a role in pre-mRNA editing, whereas ADAR1^p150^ is predominantly cytoplasmic, suggesting its main role in the cytoplasm [18,20,21]. ADAR1^p110^ was recently found to be degradaded during the early stage of type I IFN treatment (about 2–8 h) [22]. In contrast, ADAR1^p150^ is induced by type I and type II IFN [16]. Nevertheless, upon activation, both ADAR1^p110^ and ADAR1^p150^ suppress IFN expression and IFN mediated antiviral activity [22].

The close interaction between IFN and ADAR1 enables ADAR1 in controlling innate immunity, as demonstrated by the fact that *Adar1^−/−^* mice have an embryonic lethal phenotype with aberrant activation of IFN signaling, defective erythropoiesis and widespread apoptosis [23,24,25,26]. ADAR1 protects the organism from several diseases associated with IFN activation such as the autoimmune Aicardi-Goutières syndrome [27], dyschromatosis symmetrica heterditaria [28,29] and psoriasis [30]. Not surprisingly, in addition to autoimmune disease, ADAR1 is also involved in cancer immune recognition [31,32]. Notably, both editing dependent and independent mechanisms are revealed.

This review will focus on the regulatory mechanisms of the ADAR1 enzymes during tumorigenesis through aberrant editing of specific substrates or suppressing the innate immunity response. For a more extensive review on the role of both ADAR1 and ADAR2 in cancer we refer to [33].

## 2. The Role of ADAR1 in Modulating Specific Editing Substrates during Cancer Progression

*Adar1* is frequently amplified with elevated activity in many different cancer types, consistent with the elevated editing levels of its substrates [11,12]. These alternations found in cancer are mainly explained by the IFN response and gains in *Adar1* copy number [34,35]. In this way, elevated ADAR1 expression promotes cancer growth and metastasis in e.g., hepatocellular carcinoma, breast cancer, esophageal cancer, prostate cancer and multiple myeloma [36,37,38,39,40,41,42,43,44,45,46,47,48]. However, in a few cases, such as melanoma and invasive breast cancer cells, silencing or deletion of ADAR1 can also enhance the malignant properties [32,49,50,51].

Similar to somatic mutations in DNA, most RNA mutations are likely to be passengers without any effects. However, some aberrant editing events may be functionally equivalent to driver mutations, making a remarkable contribution to cancer progression and metastasis. These driving editing events could either happen within coding regions or non-coding regions. RNA editing that leads to recoding of a transcript has mainly been found to contribute to carcinogenesis through reducing the activity of tumor suppressors such as bladder cancer associated protein (BLCAP) [41,52] or enhancing the activity of pro-survival genes such as Antizyme inhibitor 1 (AZIN1) [36,37,38,39] (Table 1). RNA editing has an increasing importance in giving rise to the peptide’s heterogeneity. In an integrated analysis of both liquid chromatography tanden mass spectrometry (LS-MS) based dataset and an RNA seq dataset, Peng et al., revealed a striking 40% variation in peptides caused by RNA editing per patient sample in breast cancer [10]. Significantly more editing events are found in intronic and 3’UTR regions of pre-mRNA and non-coding RNAs [34,53,54,55], some of which also have functional consequences, for instance, alter protein expression levels (Table 1).

### 2.1. RNA Editing Dependent Role of ADAR1 in Modulating Specific Editing Substrates

AZIN1 is one of the most well-studied ADAR1 substrates in cancer, the edited form of which is strongly associated with cancer progression. This editing event is elevated in hepatocellular carcinoma (HCC) primarily due to upregulated ADAR1 expression. A-to-I RNA editing results in a serine to glycine (S/G) conversion at residue 367 in AZIN1. The edited form, AZIN1^S367G^, is more stable and has a stronger affinity to antizyme. Antizyme is known to regulate cell growth by binding and degradation of growth promoting proteins such as ornithine decarboxylase (ODC) and cyclin D1(CCND1), which are two essential factors for the G1/S cell cycle checkpoint. Compared to wild type AZIN1, AZIN1^S367G^ has greater antizyme binding, inhibiting antizyme-mediated degradation of ODC and CCND1, thereby facilitating entry into cell cycle and increasing the malignancy of the cancer cell [36]. In addition to hepatocellular carcinoma, AZIN1 RNA editing levels are also significantly elevated in esophageal squamous cell carcinoma (ESCC) [37], non-small-cell lung cancer [38] and colorectal cancer when compared with corresponding normal mucosa [39]. High levels of AZIN1 RNA editing emerged as a prognostic factor for overall survival and disease-free survival and was an independent risk factor for lymph node and distant metastasis [39].

The transcript coding for BLCAP, a tumor suppressor gene, is hyper-edited in its coding region in cervical cancer [40] and HCC [41]. Hyper RNA editing of BLCAP is positively associated with tumor size and tumor numbers, suggesting that editing of BLCAP contributes to hepatocarcinogenesis [41]. BLCAP editing increases cell proliferation by activation of the Akt/mTOR signaling pathway [41] or STAT3, a well-recognized pro-survival protein [40]. In contrast, BLCAP was found to have reduced editing levels in astrocytoma, bladder cancer and colorectal cancer, which correlated with the increase of the histological malignancy of the tumors [56].

ADAR1 mediated glioma-associated oncogene 1 (GLI1) editing leads to an R/G amino acid change at residue 701, which is significantly higher in relapsed multiple myeloma (MM) than age matched controls [42] (Table 1). GLI1^R701G^ was found to stabilize its transcriptional activity, and thus positively promote its regulated Hedgehog pathway and stem cell self-renewal [42,57]. ADAR1 knockdown significantly reduced engraftment of myeloma in vivo when performed on a serial transplantation of high risk MM in mice, supporting a vital role of ADAR1 activity in malignant regeneration of MM. Tissues with the highest GLI1 editing rates were also associated with the highest levels of human cell engraftment and ADAR1 expression. In addition, ADAR1 dependent RNA editing of GLI1 led to resistance of myeloma cells to chemotherapeutic drug lenalidomide treatment [42], suggesting that inhibition of GLI1 editing could enhance the cell sensitivity to drug treatment.

Another ADAR1 substrate identified to be hyperedited and of clinical importance in MM, as revealed by a transcriptomic analysis, is endonuclease 8-like 1 (NEIL1) [43] (Table 1). NEIL1 is a base excision repair protein that is involved in DNA damage repair. Edited NEIL1 was found to have a reduced oxidative damage repair capability, rendering cells to be more sensitive to combination treatment of single-stranded DNA breaks and double-stranded break inducing agents. Notably, in this study, they also revealed that overexpressed ADAR1 driving poor proganosis in cancer patients was independent of 1q21 status of amplification [43].

In addition to RNA editing mediated amino acid substitutions in specific genes contributing to cancer, elevated ADAR1 editing of non-coding regions or non-coding RNAs, can also promote cancer progression (Table 1). The pro-survival gene dihydrofolate reductase (*DHFR*) is edited at multiple sites within two inverted Alu repeats in the 3’-untranslated region (3’-UTR) of its transcript. RNA editing deprives DHFR from microRNA silencing, which, consequently, stabilizes its mRNA and editing levels have been shown to be higher in breast cancer compared to adjacent normal tissues [44]. The DHFR enzyme is crucial to cell proliferation due to its role in nucleotide and amino acid synthesis and is targeted by several chemotherapy agents, e.g., methotrexate that acts by preventing cancer cell division. Knockdown of ADAR1 increases cancer cell sensitivity to methotrexate by suppressing DHFR expression [44]. Another example of specific editing within the non-coding part of a gene, resulting in stabilization of its transcript, is intronic editing of the oncogene focal adhesion kinase (FAK) transcript. A-to-I editing FAK has been shown to promote lung adenocarinoma cell migration and invasion [45].

Long non-coding RNA (lncRNA) editing has also been shown to play a role in tumor development. One example is lncRNA prostate cancer antigen 3 (PCA3), which is upregulated in human prostate cancer [58] (Table 1). PCA3 is antisense to the prune homolog 2 (PRUNE2) and forms a duplex with PRUNE2 pre-mRNA. The PCA3/PRUNE2 duplex undergoes A-to-I editing at multiple sites by ADAR1, which leads to reduced levels of PRUNE2, increased PCA3 expression levels and, thereby, a subsequent increase in cancer cell proliferation, adhesion and migration [46].

Moreover, editing of microRNA affects their biogenesis or alters their target gene specificity [59], which also contributes to cancer progression. For example, RNA editing close to the DROSHA/DGCR8 cleavage site at +3 on tumor suppressor pri-let-7d impaired its biogenesis in chronic myelogenous leukemia [47] (Table 1). Editing-induced reduction of let-7d levels enhanced self-renewal of progenitors, resulting in malignant reprogramming of progenitors into blast crisis leukemia stem cells. In another study, miRNA-200b was found to be hyper-edited in a variety of cancer types and elevated editing levels were shown to correlate with poor survival in cancer patients, suggesting its prognostic role (Table 1). Edited miR-200b promoted cell invasion and migration primarily through its impaired ability to inhibit epithelial-mesenchymal transition (EMT) regulator ZEB1/ZEB2 and instead repressed a new group of targets such as leukemia inhibitory factor receptor (LIFR), a well-characterized metastasis suppressor [48].

Elevated ADAR1 expression in breast cancer has been shown in high throughput sequencing studies [12,34,60], and enhanced editing in DHFR, as described above, was found to be associated with breast cancer malignancy [44]. However, a recent study added another layer of complexity to the role of ADAR1. Gumireddy et al., found that ADAR1 protein expression was lower in metastasizing breast cancer cells as compared to non-invasive ones. The edited form of the GABA_A_ receptor subunit gamma-aminobutyric acid receptor subunit α-3 expressed from the Gabra3 transcript was only detected in non-invasive breast cancer tissue or cells but absent in invasive tumors [49]. The α-3 subunit is known to suppress tumor cell invasion and metastasis by activating the AKT pathway [61]. Editing of Gabra3 reduces its own expression on the cell surface [62], and thereby, inhibits AKT activation, suppressing tumor migration, invasion and metastasis [49] (Table 1).

Another exception to the rule, and unlike most other cancer types described above, significantly reduced ADAR1 expression has been consistently found in metastatic melanoma compared to melanocytes [50,63] (Table 1). Knockdown of ADAR1 promotes melanoma growth and metastasis by controlling the biogenesis of oncogenic or tumor suppressing miRNAs. For instance, editing of pri-miR-455-5p at the +2 and +17 sites by ADAR1 prevents drosha ribonuclease III (DROSHA) from cleaving, thus resulting in low levels of mature miR-455-5p. Therefore, miR-455-5p in its wild-type form increases tumor growth and metastasis by regulating the expression of the tumor suppressor CPEB1, but has the reverse effect in its edited form [50]. Edited miR-378a-3p at the +18 site binds to the new gained target PARVA oncogene and inhibits its expression, thereby preventing the melanoma malignancy [51] (Table 1).

Interestingly, even peptides derived from edited transcripts have been shown to function as cancer antigens that activate immune cell mediated tumor cell killing [32]. Five edited peptides (9-11 amino acids long) from three editing sites were identified to be human leukocyte antigen (HLA) ligands in a high resolution mass spectrometry based immunopeptidomics from primary human tissues. Among them, ADAR1 edited cyclin I (CCNI)^R75G^ peptides were characterized to activate tumor infiltrating lymphocytes (TIL) generated from human melanoma tumors. Moreover, CCNI^R75G^ peptides, but not wild type CCNI, facilitates TIL mediated tumor cell killing [32] (Table 1).

### 2.2. RNA Editing Independent Roles of ADAR1 in Modulating Specific Substrates

Although A-to-I editing is ADAR1’s main function, it is hard to ignore the editing-independent role of ADAR1 as a dsRNA binding protein. Different possible mechanisms mediated by ADAR1 have also been revealed to play a role in carcinogenesis. In some of the studies, ADAR1 were found to regulate specific targets at the transcriptional- and post-transcriptional level and thereby inhibit cancer progression [31,64]. Tumor cells, particularly melanoma cells, has been demonstrated in these studies to downregulate ADAR1 expression in order to survive and migrate.

In one recent study, Nemlich et al., discovered a novel role of ADAR1 regulating melanoma growth by negatively controlling integrin beta 3 (ITGB3) at both transcriptional and post-transcriptional levels, via transcription factor PAX6 and miR-22, respectively. ITGB3 is a cell surface protein that was previously found to be correlated with aggressiveness of the tumors [65,66,67]. Silencing of ADAR1 in melanoma cells upregulated ITGB3 expression, thus promoting melanoma malignancy [64]. In another study, Galore Haskel et al., found that ADAR1 knockdown enhances the biogenesis of miR-222 and thereby suppresses the expression of the miR-222 targeted mRNA, intercellular adhesion molecule 1 (ICAM1) [31]. ICAM1 is one of the natural ligands for lymphocyte function associated antigen-1 (LFA-1) and expressed on most leukocytes [68]. Upon binding to LFA-1, ICAM1 helps the formation of the immune synapse [69] and activates T cells [70]. Overpression of ADAR1 in melanoma cells enhances ICAM1 expression, which therefore renders tumor cells more sensitive to TIL mediated killing [31]. Moreover, two groups of miRNA expression profiles were compared in between metastatic melanoma tissue specimens derived from patients that showed clinical benefit after ipilimumab treatment and those who did not. Ipilimumab is an immune checkpoint inhibitor that blocks the cytotoxic T-lymphocyte associated protein 4 (CTLA-4) [71]. Strikingly, miR-222 was found to be the only one out of over one thousand of miRNA that showed significant differential expression between the two groups, indicating that miR-222 might be a potential marker to predict melanoma patients reponse to the immunotherapheutic drug ipilimumab [31].

## 3. A Possible Role of ADAR1 as an Immune Repressor during Cancer Progression

ADAR1 is responsible for editing at many specific sites, but none of them can explain the embryonic lethality outcomes of the absence of ADAR1 in mice [23,24]. However, the main ADAR1 target in human are inversely oriented Alu repeats forming long dsRNA stem-loop structures [53,54,55,72]. The absence of editing within these structures leads to increased levels of cellular dsRNA, which trigger an IFN signaling response [73]. Indeed, the aberrant immune response was suppressed by I:U dsRNA transfection in mouse embryonic fibroblasts with mutant ADAR1 [74].

**Figure 1:**
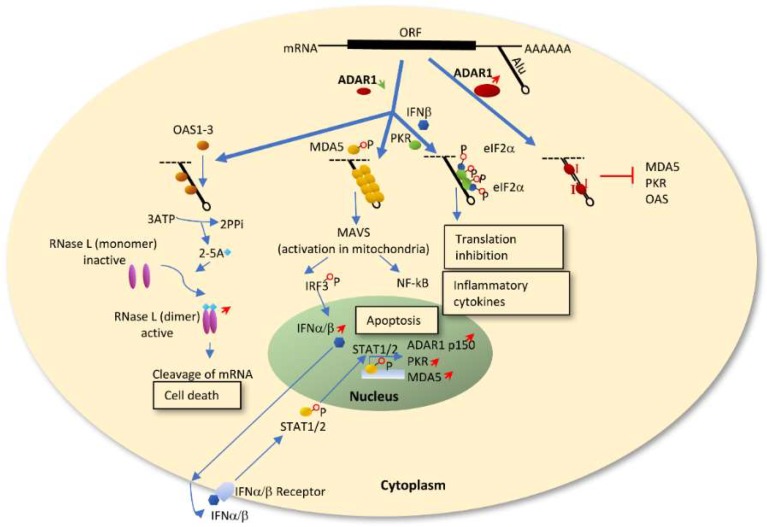
Proposed three innate immune response signaling pathways regulated by ADAR1. When ADAR1 is silenced or its editing activity is abolished, the endogenous promiscuous dsRNAs, mainly Alu repeats, remain paired and serve as substrates for MDA5, OAS and PKR, enabling the activation of the corresponding downstream pathways. ADAR1 mediates A-to-I editing of dsRNA and prevents MDA5/PKR/OAS from sensing the partly unpaired dsRNA.

ADAR1 deficiency leading to IFN production and upregulation of IFN stimulated genes (ISGs) has been widely studied in a variety of cell types such as immune cells, macrophages, stem cells and fibroblasts [25,74,75,76,77,78]. ADAR1 modulates innate immunity primarily through the RLR-initiated cytosolic dsRNA-sensing signaling pathway[26,73,74,75,76]. During normal conditions, ADAR1 mediated A-to-I editing on promiscuous dsRNA, such as Alu repeats, prevents MDA5 sensing of the unpaired dsRNA [73,74,75]. When ADAR1 is silenced or its editing activity is abolished, the endogenous dsRNAs remain paired and serve as substrates for MDA5 to bind and form filaments [26,73,79], enabling the activation of the MAVS-IRF3 and NF-kB pathways as well as downstream IFN signaling (Figure 1). IFNα and IFNβ then trigger STAT1/2 induced apoptosis and transactivate PKR, MDA5 as well as ADAR1^P150^ which is induced late in the IFN response. Another possible dsRNA-sensing signaling pathway is via oligoadenylate-synthetase (OAS)-RNase L, which was recently discovered to be regulated by ADAR1 in cell lines. Endogenous dsRNA activates the OAS isoform OAS1-3, which produces 2’,5’-oligoadenylates (2-5A). This activates RNase L to degrade viral and host single-stranded RNAs leading to cell death [80]. ADAR1 knockdown has also been shown to activate another ISG, protein kinase R (PKR, also known as EIF2AK2), in presence of IFN or a viral infection. Triggered by dsRNA, PKR dimerizes and is autophosphorylated providing a site for eIF2a binding and translational shutdown [81,82,83] (Figure 1). In summary, ADAR1 has a dual protective role in regulating an innate immune response. On one hand, ADAR1 protects the organism from unwanted MDA5-MAVS mediated IFN production. On the other hand, during the response to IFN, ADAR1 safeguards cells from translational shutdown and cell death by preventing activation of PKR or OAS-RNase L.

Thus far, we have discussed how ADAR1 edits endogenous Alu repeats forming long dsRNA and thereby preventing a cellular innate immunity reponse. Nevertheless, although most evidence suggests that the RNA editing function of ADAR1 is required to prevent an innate immune response, editing independent mechanisms have also been suggested [75,77]. For example, in the study by Yang et al., they showed that preserving the RNA binding activity of ADAR1 but not the editing activity rescued IFN production by Sendai virus infection in the cells [77].

Although it has not yet been well established, ADAR1’s role as an immune repressor could be utilized by tumor cells for immune escape [31,32]. IFNs are induced by various cell types in the tumor microenvironment, where they help the innate immune system to recognize tumor cells at an early stage and induce the expression of both MDA5 and PKR [81]. An increase in A-to-I hyper-editing of endogenous long dsRNA by elevated ADAR1 expression may thereby hide the cancer cells from being recognized by the MDA5 and PKR pathways as they will no longer recognize these as substrates. At a later stage, tumor cells could also suppress their intrinsic secretion of type I IFN for successful metastasis [84]. Increased IFN signaling by ADAR1 inhibition thus have a positive effect in facilitating immune cells to kill tumor cells. In a recent study where immunohistochemistry was performed in a cohort of 681 breast cancer biopsies, ADAR1 expression positively correlated with tumor infiltrating lymphocyte levels as well as IFN related protein expression, including human leukocyte antigen HLA-ABC, PKR and MxA [85]. Additionally, ADAR1 was recently found to be one of the top candidades of depleted genes in a large genetic screen that increased the efficacy of immunotherapy with PD-1 checkpoint blockade in mice [86].

## 4. Clinical Application of ADAR1 Editing in Cancer

We have discussed the functions of ADAR1 in causing RNA mutations and preventing an immune response, two aspects that could serve as significant forces that drive transition from normal cell to malignant tumor. However, whether enhanced or reduced ADAR1 contributes to tumor growth largely depends on tumor types and/or target gene specificity. When considering clinical application of the editing events, different aspects should be considered. Firstly, ADAR1 expression levels as well as editing levels at specific sites may serve as prognostic biomarkers in certain cancer types, owing to their significant correlation with overall survival of cancer patients [11,12,42,48,87]. Secondly, editing levels at specific sites have been found to significantly correlate with drug sensitivity. For example, the editing in AZIN1, DHFR and GLI1 decreased cell sensitivity to chemotherapheutic drugs [12,42,44]. Thus, inhibiting editing at specific sites combined with corresponding drug treatment could provide a synergetic effect for eliminating tumor cells. Thirdly, several RNA editing sites in proteins such as AZIN1^S367G^ and GLI1^R701G^ appear to play essential driving roles in tumor progression [36,42], implying their utility as potential therapeutic targets in patients who have these RNA mutations.

The immunoregulatory effect and anti-proliferation property of IFN make it a capable therapeutic drug against cancer. However, tumor resistance to the effects of IFN greatly limits its utilization. It has been recognized that one of the reasons for tumor resistance to IFN cancer therapies is defects of IFN signaling such as loss of the ISG STAT1 [88,89]. Cells with impaired activity of transcription factor STAT1 do not respond to IFN treatment, nor is their growth inhibited [89]. ADAR1 knockdown leads to an upregulation of STAT1 [25,81], which could thus enhance the sensitivity of tumors to IFN treatment. Applying an ADAR1 inhibitor combined with IFN therapy in cancer patients could therefore lead to cell growth arrest or cell death, thereby enhancing the efficacy of IFN therapy and provide a possible promising treatment. In cancers with elevated ADAR1 expression, a specific ADAR1 inhibitor that decreases hyper-editing of long dsRNAs such as the Alu inverted repeats, may also facilitate a tumor-specific innate immune response. Our knowledge of the role of ADARs in cancer has expanded a lot in the past 18 years. Hopefully, in the next decade, ADAR as a target in cancer therapy will become a reality.

## Figures and Tables

**Table 1:** Adenosine deaminase acting on RNA 1 (ADAR1) editing of specific substrates that associate with cancer development.

Edited Form Drives Tumor Growth and Metastasis				
Gene	Protein	Edited Residues	Cancer Types	Ref.
*AZIN1*	Antizyme inhibitor 1	S/G	hepatocellular carcinoma	[36]
			esophageal squamous cell carcinoma	[37]
			non-small-cell lung cancer	[38]
			colorectal cell carcinoma	[39]
*BLCAP*	Bladder cancer associated protein	Y/C; Q/R; K/R	cervical cancer	[40]
		Y/C	hepatocellular carcinoma	[41]
*GLI1*	Glioma-associated oncogene 1	R/G	multiple myeloma	[42]
*NEIL*	Endonuclease 8-like 1	K/R	multiple myeloma	[43]
*DHFR*	Dihydrofolate reductase	3’UTR	breast cancer	[44]
*FAK*	Focal adhesion kinase	Intron	Lung adenocarcinoma	[45]
*PCA3*	Prostate cancer antigen 3	Multiple sites when forms duplex with PRUNE2	prostate cancer	[46]
*pri-let-7d*		+3; +59	chronic myelogenous leukemia	[47]
*miR-200b-3p*		+5	HNSC, KIRP, THCA, and UCEC *	[48]
**Edited Form Inhibits Tumor Growth and Metastasis**				
**Gene**	**Protein**	**Edited Residues**	**Cancer Types**	**Ref.**
*Gabra3*	Gamma-aminobutyric acid receptor subunit alpha-3	I/M	breast cancer	[49]
*miR-455-5p*		+2; +17	melanoma	[50]
*miR-378a-3p*		+18	melanoma	[51]
*CCNI*	Cyclin I	R/G	melanoma	[32]

* HNSC: Head and neck squamous cell carcinoma; KIRP: Kidney renal papillary cell carcinoma; THCA: Thyroid carcinoma; UCEC: Uterine corpus endometrial carcinoma; miR: microRNA.
